# A Scoping Review: Mapping the Evidence for Undergraduate Concussion Education and Proposing the Content for Medical Student Concussion Teaching

**DOI:** 10.3390/ijerph19074328

**Published:** 2022-04-04

**Authors:** Nick Gardner, Neil Heron

**Affiliations:** 1 Faculty of Medicine, Health and Life Sciences, Queen’s University Belfast, Belfast BT9 7BL, UK; n.gardner@qub.ac.uk; 2 Centre for Public Health, Queen’s University Belfast, Belfast BT12 6BA, UK; 3 School of Medicine, Keele University, Newcastle ST5 5BG, UK

**Keywords:** concussion, education, medical student, knowledge, scoping review

## Abstract

Introduction: Concussion is a common yet complex condition, with each new case requiring assessment by a medical doctor. Recent research has shown that doctors working in the UK have significant knowledge deficits regarding concussion diagnosis and management. Aim: The aim of this scoping review was to map out the evidence about how undergraduate medical students are being educated about concussion. Method: This scoping review involved seven research papers identified by searching five online databases in October 2020. Search terms relevant to concussion included: brain injuries, post-concussion syndrome, brain concussion and concussion, combined with search criteria for undergraduate education: medical students, undergraduate medical education, or curriculum. Results: All seven papers were published in North America, with five papers recruiting medical students from single institutions (*n* = 590) and two papers surveying universities. Canadian medical schools have shown an upward trend in the quantity of teaching about concussion-specific teaching: from 0.57 to 2.65 h between 2012 and 2018. Lectures were the commonest mode of delivery of teaching, followed by problem-based learning and clinical rotations. The studies reach a common conclusion that medical students are not being adequately prepared for diagnosing and managing concussion, with insufficient undergraduate teaching, particularly exposure during clinical rotations, cited as the cause. Conclusions: Concussion: education of medical students is inadequate in North America. Medical schools should help address this by providing lectures and clinical presentations on concussion to learn from, particularly via problem-based learning. There is a paucity of evidence about concussion education in other geographical areas.

## 1. Introduction

Twenty years ago, a consensus definition of concussion was first agreed. Deeming previous descriptions inadequate, Aubry et al. defined concussion as “a complex pathophysiological process affecting the brain, induced by traumatic biomechanical forces” [1]. The subsequent two decades have seen four revisions of this Consensus Statement on concussion in sport [2] and substantially increased media attention around this diagnosis [3], particularly with the linkage to long term neurological sequelae, including dementia syndromes.

The incidence of traumatic brain injuries, including concussions, is high and rising [4,5]. The Centres for Disease Control and Prevention (CDC) estimates that between 2002 and 2006, head trauma was the cause of approximately 1.7 million hospital attendances per year in the USA; factoring in the episodes occurring that do not result in hospital presentation, this number increases to 3.8 million [4]. Increasing incidence of concussion has also been shown through epidemiological studies, although this finding is likely to be attributable to improving knowledge and awareness of concussion, rather than an increase in concussive episodes [5]. Concussion is also a common presentation in the UK and is the reason for 6.6% of all Emergency Department presentations [6].

It is important to bear in mind the serious acute and chronic complications of this condition, and the associated responsibility that health professionals have in its timely diagnosis. The current Consensus Statement on concussion in sport states that concussion “is considered to be among the most complex injuries in sports medicine to diagnose, assess and manage” [2]. Furthermore, there is no reason to assume that the difficulties in diagnosing this “invisible injury” [7] apply only to physicians working in sports medicine. This Consensus Statement highlights that there is “no perfect diagnostic test” and therefore the assessing physician must have a low threshold for completing a thorough examination for symptoms and signs of concussion [2].

In addition to being a common presentation, concussion can potentially lead to serious and disabling sequelae if not diagnosed immediately [8]. In a situation where subsequent head trauma is possible, such as during sport participation, there is an acute risk of second impact syndrome (SIS), which has caused a number of high profile deaths in athletes. Second impact syndrome is a known complication of repeated head trauma which occurs when “an athlete who has sustained an initial head injury, most often a concussion, sustains a second head injury before symptoms associated with the first have fully cleared” [9]. Although its incidence is extremely rare, children and adolescents are at a higher risk of developing SIS and the consequences can be devastating, with mortality rates approaching 50% [10]. As such, educational campaigns (for example “If in doubt, sit them out”) have sought to raise awareness among players and coaches involved in sport about the importance of removing players from the field of play if there is any concern about concussion [8].

Educational programmes targeting those involved in recreational sports have been shown to be effective in increasing awareness and understanding around concussion. Since 2003, the CDC based in America has been running the “Heads Up” campaign with the aim of improving prevention, recognition and response to concussion. Coaches involved in youth sports have reported a positive response to this: 77% of respondents report being better at identifying athletes who may be concussed, 63% were more aware of the seriousness of the condition, and 50% had learned something new about concussion after reading the “Heads Up” material [11].

Concussion should certainly be considered as a diagnosis that all graduating medical students need to be ready to investigate and manage. Research has shown that all concussed athletes should be assessed by a medical doctor [7]. Cases of concussion can present to doctors working in a variety of fields, including General Practice, Emergency Medicine and General Medicine including Neurology, Paediatrics and Sports Medicine [12,13]. Ropper and Gorson, extrapolating from the high incidence rate of this condition, conclude that “almost all physicians are called on at some time to provide care at the scene or to treat the sequelae of concussion” [14]. This information needs considering in the context of recent research which has shown that doctors working in postgraduate training in the UK may have significant knowledge deficits regarding concussion diagnosis and management [15].

To the present writer’s knowledge, to date, there have not been any scoping reviews conducted into concussion education of undergraduate medical students. The aim of this project, therefore, is to map the recent evidence about how undergraduate medical students are being taught about concussion.

## 2. Methodology

This scoping review was based on the six-step methodological framework developed by Arksey and O’Malley [16] and Levac et al. [17]. The review was conducted in accordance with the Preferred Reporting Items for Systematic Reviews and Meta-Analysis (PRISMA) extension for scoping reviews [18]. The Prisma extension for Scoping Reviews (PRISMA-ScR) checklist was completed [18,19]. The aim of this review was to thematically present all the data available on undergraduate concussion education for wider reading, and in doing so inform a proposal for medical student teaching. A secondary aim was to highlight areas of further research needed to more accurately implement any necessary changes in medical student teaching.

The research topic was proposed by NH. NH acted in a supervisory role, with NG carrying out the initial searches. Relevant research papers were identified using five online databases: Medline, Scopus, Embase, Pubmed, Web of Science and Google Scholar. Database searches were carried out in October 2020. The following search terms were used to identify publications relevant to concussion: brain injuries, post-concussion syndrome, brain concussion and concussion. These search terms were subsequently combined with search criteria for undergraduate education: medical students, undergraduate medical education, or curriculum.

The authors of the most recent Consensus Statement on concussion in sport refer to this as an “unresolved issue”, stating that it is unclear whether concussion is accepted on the milder end of a TBI spectrum, or whether its symptoms and signs are due to reversible physiological changes [2]. Whilst the acronym “mTBI” was not added as an additional search term on the databases above, this was a term kept in mind when analysing the titles of papers identified with the search criteria above.

Given that one aim of this project is to present recent data on the state of undergraduate education, and that the medical knowledge around concussion has been quickly evolving, papers from 2010 onwards more accurately reflect current day knowledge and practice of concussion management. As a result, only papers published from the start of 2010 onwards were included. Only full-text papers were included; where only abstracts were available, these were excluded unless sufficient data was presented to contribute to meaningful results [20]. No geographical exclusions were implemented. However, papers not published in English were excluded.

**Figure 1:**
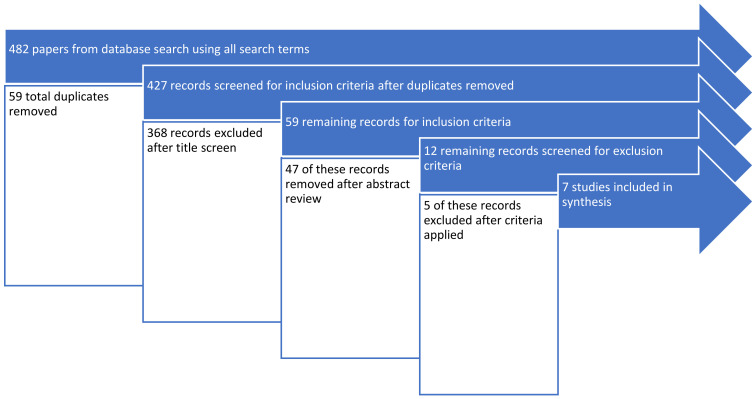
Exclusion process of papers.

At this stage of the search process, 482 papers met the above inclusion criteria entered into the search databases, of which 55 papers were duplicates and subsequently removed (see Figure 1). After review by NG and NH, this number was reduced to 59 papers by examination of the titles and abstracts, and subsequently to 12 papers after reviewing the full-text articles. The initial exclusion of papers was based on the title clearly indicating that it was not related to undergraduate medical education or concussion. In the remaining 59 papers, abstracts were read to determine suitability for inclusion. A further 47 papers were excluded at this stage. The reasons for excluding papers are presented in Table 1.

The remaining 12 papers had full-text reviews by both researchers, NG and NH and five further papers were excluded at this stage: three were deemed not specific to concussion and focused on more generalised trauma presentations, one publication was a duplication (of a preliminary publication on the same data) of a paper already included in the study, and a further paper was not primary research. There was complete agreement between reviewers about the exclusion of these papers, resulting in a final search outcome of seven papers that met the inclusion criteria.

Once the final group of papers had been established, it was possible to consider the data charting process. Common themes present in two or more papers enabled data to be compiled and the formulation of six questions to be addressed:

1. Where has research been carried out?
2. When were the studies published?
3. What methodology was used?
4. How much time is dedicated to undergraduate teaching about concussion?
5. How is undergraduate concussion education being delivered?
6. How effective is undergraduate concussion education?

## 3. Results

Seven papers were included in this scoping review: all original research papers involving undergraduate medical students and published between 2010 and 2020 in English. The published papers showed a geographical predilection to North America and Canada: three papers published from the former and the remaining four in the latter. Only papers published in the past decade were included in this project. With a paper published every year for the past five years, this may suggest an upward trend in interest in this research. All seven papers meeting the inclusion criteria used questionnaires to assess concussion education. The methodologies of the seven papers are summarised in Table 2. For five of these papers, medical students were the target of the survey, whilst two studies enlisted university deans or their educational representatives to complete surveys. Two papers compared results following an educational intervention.

Three of the seven papers forming this scoping review gave details about the amount of time assigned for concussion teaching [3,21,22]. In 2012, Burke et al. [21] found that Canadian universities on average dedicated a little over 30 min of formal concussion teaching to their undergraduates, and just over 90 min of general head injury teaching with a concussion component. An almost identical survey in 2017 [3] revealed that teaching quantity had increased significantly: all the responding medical schools reported that they provided head injury education incorporating a concussion component, with 11 of these schools teaching concussion-specific education. The amount of time dedicated to concussion-specific education had increased to 2.65 h and time devoted to head injury teaching with a concussion component was found to be 7.5 h, both findings almost five times the amount found in 2012. A third paper quantified the concussion teaching at the undergraduate level [22], and also showed an upward trend in the quantity of teaching time. Following a redesign of the curriculum in 2016, the number of hours of concussion-specific teaching increased from 3.95 h to 10.8 h. No papers outside Canada have quantified the amount of concussion teaching undergraduate medical students are receiving.

**Table 2:** The subject of questionnaires in included research papers.

Paper	Year	Country	Subject Surveyed	Number	Questionnaire Contents	Intervention?	Type of Intervention	Appraisal of Methodology and Potential Biases
Burke, M. J., Chundamala, J., and Tator, C.H. [21]	2012	Canada	University deans or designated representatives.	14	Amount and delivery of education.	No	n/a	Potential responder bias; provides information on duration of teaching but does not assess students’ knowledge; not independently verified responses.
Boggild, M and Tator, C. [23]	2012	Canada	Fourth-year medical students at a single institution.	52	Questions about concussion.	No	n/a	Not a previously validated questionnaire, used international guidelines and pilot tested; represented 23% of medical school year; non-examination conditions.
Donaworth, M.A., Grandhi, R.K. et al. [12]	2016	USA	First–fourth-year medical students at a single institution.	266	Questions about concussion adapted from [23].	No	n/a	Large sample size; even spread over 4 years; potential responder bias, one third response rate.
Haider, M. N., Leddy, J.J., et al. [13]	2017	USA	Medical students rotating in sports medicine at a single institution.	9	MCQs about concussion and delivery of undergrad. teaching.	Yes	Literature vs Literature + clinical exposure.	Small sample size; potential selection bias of participants; not validated survey.
Mathieu, F., Ellis, M.J., and Tator, C.H. [3]	2018	Canada	University deans or designated representatives.	13	Amount and delivery of education, + changes from 2011.	No	n/a	Potential responder bias; provides information on duration of teaching but does not assess students’ knowledge; not independently verified responses.
Fraser, S., Wright, A. D., et al. [22]	2019	Canada	Medical students: 1st years compared with 2nd–4th years at a single institution.	148	Questions about concussion adapted from [23] and [12].	Yes	Change in curriculum from block to spiral.	Excluded participants that lacked making an “honest effort”; late responders analysed; low response rate of 12.5%.
Holschen, J.C., and Benert, J. [20]	2020	USA	First- and fourth-year medical students at a single institution.	115	Questions about concussion.	No	n/a	Not validated survey; high response rate of 71%. Abstract only.

All seven papers provide at least some data on how undergraduate education is being delivered. Burke et al. [21] and Mathieu et al. [3] identified that in Canadian universities, lectures were the most common delivery format, followed by problem-based learning (PBL) and clinical rotations. Fraser et al. [22] based at the University of British Columbia reports that prior to 2016, concussion education was divided approximately equally between lectures and PBL. Following a review of the curriculum, problem-based learning became the predominant format, with 7.5 h of PBL compared with 3.3 h of lectures. The remaining four papers [12,20,23] collected data from medical students about how they were educated on concussion. These papers give a similar description of the education provided: lectures are reported as the commonest mode of delivery, with clinical rotations (particularly Emergency Medicine) forming another important (or at least memorable) learning opportunity. It is important to note that between 8% [23] and 38% [12] of medical students reported never being taught about concussion and between 16.8% [12] and 24% [23] had no memory of receiving teaching.

Five studies have sought to quantify medical students’ knowledge about concussion [12,13,20,22,23]. Three papers surveying medical students’ knowledge to identify and quantify deficits in concussion knowledge will be outlined first, while two papers sought to quantify knowledge in the contexts of educational interventions. Holschen and Benert [20] examined 115 first- and fourth-year medical students about their knowledge of concussion diagnosis and management. Just six first-year and thirteen fourth-year students passed the test (scoring ≥ 75%) causing the authors to conclude that medical students have “insufficient knowledge to properly assess, advise and manage patients with concussion” [20]. Donawarth et al. [12] demonstrated that deficits were maintained throughout medical school; 266 medical students completed a survey designed to explore their knowledge about concussion. The authors concluded that the “lack of exposure to education and hands-on training may contribute to knowledge deficits”. A third study [23] that surveyed medical student knowledge about concussion provides further support to the supposition that medical students are being inadequately prepared for diagnosing and managing concussion. The medical students included in this study were all in their final year of training, and yet the mean score in the survey was less than 50%.

Fraser et al. [22] found that despite a change in the curriculum there were still deficiencies in the understanding of diagnosis, with over half of the students in both curricula incorrectly answering questions about diagnostic criteria. Lastly, it is worth mentioning that, although the number of recruited medical students was too small to be representative, Haider et al. [13] were able to demonstrate a statistically significant improvement in test scores in medical students who were given exposure to concussion through clinical rotations.

## 4. Discussion

This scoping review compiled data from seven papers published since 2010. Each study carried out primary research into how undergraduate medical students are being taught on the topic of concussion. All participants were enrolled in North American universities. In total, 590 medical students, and at least 14 of the 17 Canadian medical schools, are represented in this scoping review. Lectures were the commonest teaching format, although clinical rotations, particularly in Emergency Medicine and Neurology, also provided learning opportunities. Overall, knowledge on concussion appears to be poor, with the majority of studies concluding that medical students demonstrate significant educational gaps.

One finding that must be highlighted is that a significant proportion of medical students are not receiving any education about concussion throughout their entire undergraduate training. Burke et al. [21] found that in 2012, four out of fourteen universities were not dedicating any time to teaching about concussion, and whilst all of the thirteen institutions responding to the same question in 2018 [3] reported providing some education, four medical schools failed to respond to this questionnaire. This leaves questions unanswered about whether these non-responding universities are less likely to be providing formal teaching about concussion. Data from four other papers [12,13,20,23] found that between 10% and 38.4% of medical students in additional institutions reported that they had not received any teaching on this topic. This is a worrying finding. As previously discussed, cases of concussion can present to almost all medical specialities. In the UK, foundation programme doctors will be independently diagnosing and treating patients in General Practice and Emergency Departments one year after graduation. We have already seen that concussions commonly present to these specialities. It is essential that these doctors are being adequately prepared to manage these cases.

Another important finding is that, although the amount of time allotted to concussion education increased during this five-year period, the delivery format did not change significantly. Provvidenza and Johnston [7] carried out a qualitative literature review into knowledge transfer and concussion education in 2009. They sought to identify effective means of communicating knowledge about concussion to healthcare professionals. The results of this study showed that didactic lectures and educational literature had little impact on changing physicians’ performance. Interactive educational sessions were effective in helping physicians apply current knowledge, as was educational outreach events (visits by educators). Whilst the participants of this review were postgraduate, and the intention behind the educational method was to update rather than teach from no prior knowledge, its specificity to concussion increases the findings’ applicability here. This scoping review has found evidence of a shift in delivery format away from lectures to PBL/case-based learning and clinical rotations, with evidence to suggest that seeing patients with concussion may help to improve knowledge deficits [13].

An almost consensus conclusion from the papers included in this scoping review was that medical students are not being adequately educated on concussion. This conclusion implies an urgent need for institutions to ensure that their medical students are being given sufficient opportunities for clinical exposure to concussion, and to rigorously evaluate how knowledgeable their undergraduates are about this important topic. Mathieu et al. [3] report that some universities appear to consider concussion a topic to be learned as a postgraduate, and not a condition that all medical graduates need to be aware of. This paper concluded that some institutions must perceive concussion education as “less relevant to the generalist physician and better suited to residency training programs in Emergency Medicine or neuroscience-related fields”. The authors echo the rationale expressed above that all physicians need to be able to diagnose and manage concussion and describe deferring concussion education to postgraduate training as “risky”.

The findings of this project lead to several recommendations. Firstly, medical schools need to ensure that all medical undergraduates are being provided with concussion-specific education because there is evidence to suggest that a significant proportion of medical students are not receiving any teaching on this condition. Secondly, the delivery format of concussion education should be reviewed, with an emphasis on PBL or case-based learning and clinical exposure, in addition to lectures, considered suitable learning methods. Thirdly, medical students should be encouraged to take advantage of the opportunities for learning about concussion during clinical rotations, particularly in Primary Care and Emergency Medicine. Future research projects would serve to reinforce these suggestions with more evidence. Research is needed over a wider geographical area to describe the landscape of concussion education in the UK and Europe. More research is also needed to answer questions about how best to deliver concussion education to medical undergraduates, and the amount of teaching time that is needed to provide medical students with the knowledge they need to safely diagnose and manage concussion.

## 5. Strengths and Limitations

Seven primary research papers were included in this scoping review in order to map all the available evidence about undergraduate medical education. Five independent research databases were searched, in addition to searching Google Scholar, in an effort to ensure, as far as is possible, that all papers meeting the inclusion criteria were represented. Two researchers (NG and NH) independently reviewed the papers, and a consensus was reached about the final seven papers. The papers included were critically appraised using the JBI Critical Appraisal Checklist, which guided the details included in Table 2 [24]. One of the papers included in the scoping review was only available as an abstract. Data presented in the abstract contributed to the findings in this scoping review but the limited information available greatly limited a rigorous critical appraisal of the methodology and analysis of the authors’ conclusions. Two papers, of which only abstracts were available, had to be excluded because they did not contain sufficient information about medical students to contribute to the findings of this scoping review. One of the included papers contained data on a sample size of only nine medical students, which the authors concluded was too small a group to give an accurate representation.

## 6. Conclusions

Concussion is a common and complex condition that all graduating medical students need to be familiar with. There is a strong likelihood that all physicians will be required to assess and manage a patient with concussion at some stage in their career, regardless of the area of medicine they choose to specialise in. Recent research from North America suggests that medical students are not being adequately prepared to treat patients presenting with concussion. Insufficient teaching time, in particular a lack of exposure during clinical rotations, was identified as a likely contributor to this finding. However, there is some evidence to suggest that medical students’ knowledge about concussion can be improved with increased teaching time, through a change in teaching format and by increasing clinical exposure.

## Figures and Tables

**Table 1:** Papers excluded at abstract stage.

Postgraduate population.	23
Allied health professional population.	10
Not specific to concussion education.	3
Not primary research.	5
2009 or earlier publication date.	5
Abstract only. No data available for medical students.	1
**Total**	**47**

## Data Availability

All data are freely available within the publication.
